# Intractable duodenal ulcer caused by transmural migration of gossypiboma into the duodenum - a case report and literature review

**DOI:** 10.1186/1471-2482-14-36

**Published:** 2014-06-10

**Authors:** Yun-Xiao Lv, Cheng-Chan Yu, Chun-Fang Tung, Cheng-Chung Wu

**Affiliations:** 1 Department of Hepatobiliary Surgery, Dongyang People’s Hospital, No60,West Wuning Road, Dongyang, Jinhua, Zhejiang, China; 2 Department of Surgery, Taichung Veterans General Hospital, 1650 Taiwan Boulevard Sect. 4, Taichung, Taiwan; 3 Department of Internal Medicine, Taichung Veterans General Hospital, 1650 Taiwan Boulevard Sect. 4, Taichung, Taiwan

**Keywords:** Gossypiboma, Duodenal ulcer, Transmural migration, Surgical complication, Duodenorrhaphy, Endoscopy, Endoscopic extraction

## Abstract

**Background:**

Gossypiboma is a term used to describe a mass that forms around a cotton sponge or abdominal compress accidentally left in a patient during surgery. Transmural migration of an intra-abdominal gossypiboma has been reported to occur in the digestive tract, bladder, vagina and diaphragm. Open surgery is the most common approach in the treatment of gossypiboma. However, gossypibomas can be extracted by endoscopy while migrating into the digestive tract. We report a case of intractable duodenal ulcer caused by transmural migration of gossypiboma successfully treated by duodenorrhaphy. A systemic literature review is provided and a scheme of the therapeutic approach is proposed.

**Case presentation:**

A 61-year-old Han Chinese man presented with intermittent epigastric pain for the last 10 months. He had undergone laparoscopic cholecystectomy conversion to open cholecystectomy for acute gangrenous cholecystitis 10 months ago at another hospital. Transmural migration of gossypiboma into the duodenum was found. Endoscopic intervention failed to remove the entire gauze, and duodenal ulcer caused by the gauze persisted. Surgical intervention was performed and the gauze was removed successfully. The penetrated ulcer was repaired with duodenorrhaphy. The postoperative period was uneventful.

We systematically reviewed the literature on transmural migration of gossypiboma into duodenum and present an overview of published cases. Our PubMed search yielded seven reports of transmural migration of retained surgical sponge into the duodenum. Surgical interventions were necessary in two patients.

**Conclusion:**

Transmural migration of gossypiboma into the duodenum is a rare surgical complication. The treatment strategies include endoscopic extraction and surgical intervention. Prompt surgical intervention should be considered for emergent conditions such as active bleeding, gastrointestinal obstruction, or intra-abdominal sepsis. For non-emergent conditions, surgical intervention could be considered for intractable cases in which endoscopic extraction failed.

## Background

Gossypiboma is a term used to describe a mass that forms around a cotton sponge or abdominal compress accidentally left in a patient during surgery. Transmural migration of an intra-abdominal gossypiboma has been reported to occur in the stomach, duodenum, ileum, colon, bladder, vagina and diaphragm [1-3]. Open surgery is the most common approach in the treatment of gossypiboma. However, gossypibomas can be extracted by endoscopy while migrating into the digestive tract. We report a case of intractable duodenal ulcer caused by transmural migration of gossipyboma successfully treated by duodenorrhaphy. We systematically reviewed the literature on transmural migration of gossypiboma into duodenum and present an overview of published cases. A scheme of the therapeutic approach is also proposed.

## Case presentation

**Figure 1:**
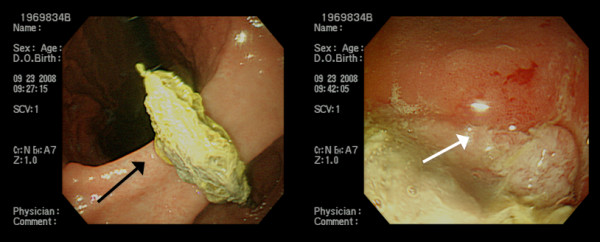
**Endoscopic findings of the duodenum.** Endoscope shows a sponge in the duodenum (black arrow) and the white-based ulcer around the gauze (white arrow).

**Figure 2:**
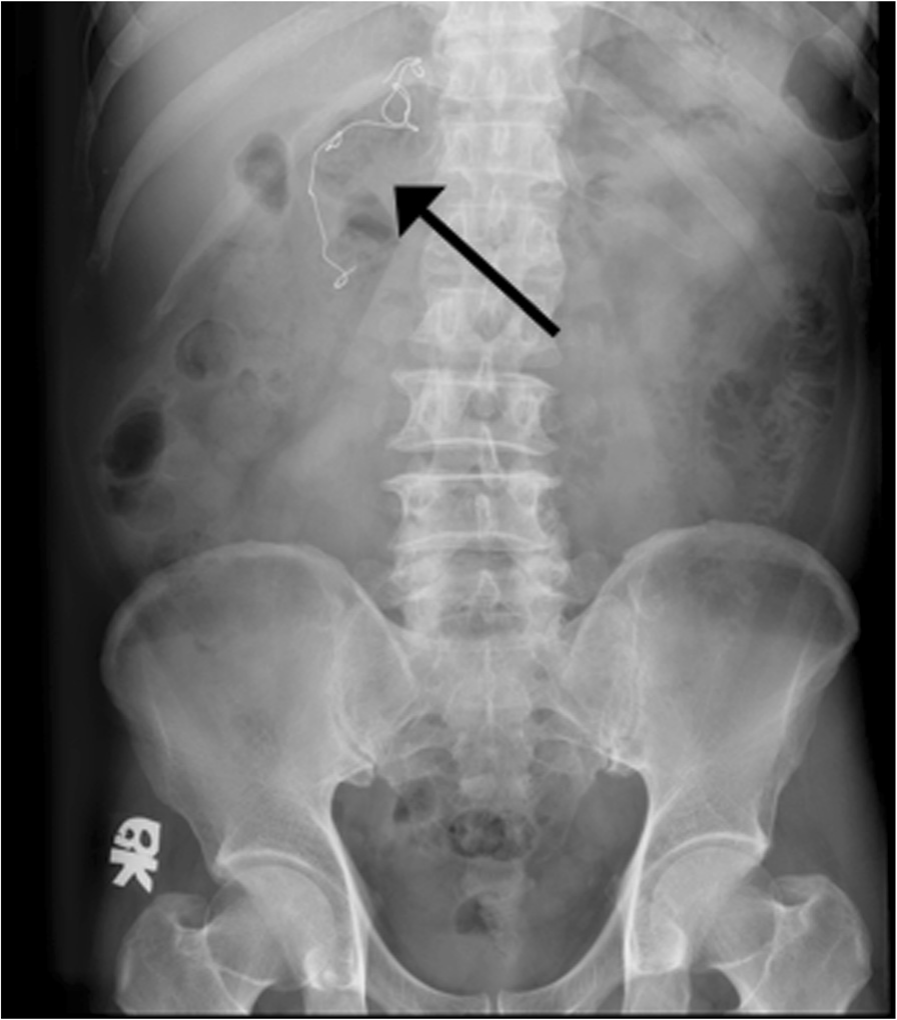
**The plain film radiograph.** Plain abdominal X-ray shows a retained surgical gauze (black arrow) in the abdominal cavity.

**Figure 3:**
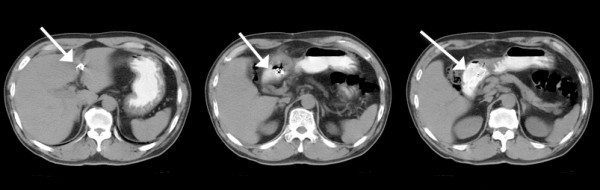
**Abdominal CT scan.** The CT-scan reveals transmural migration of the gauze into the duodenum (white arrow).

**Figure 4:**
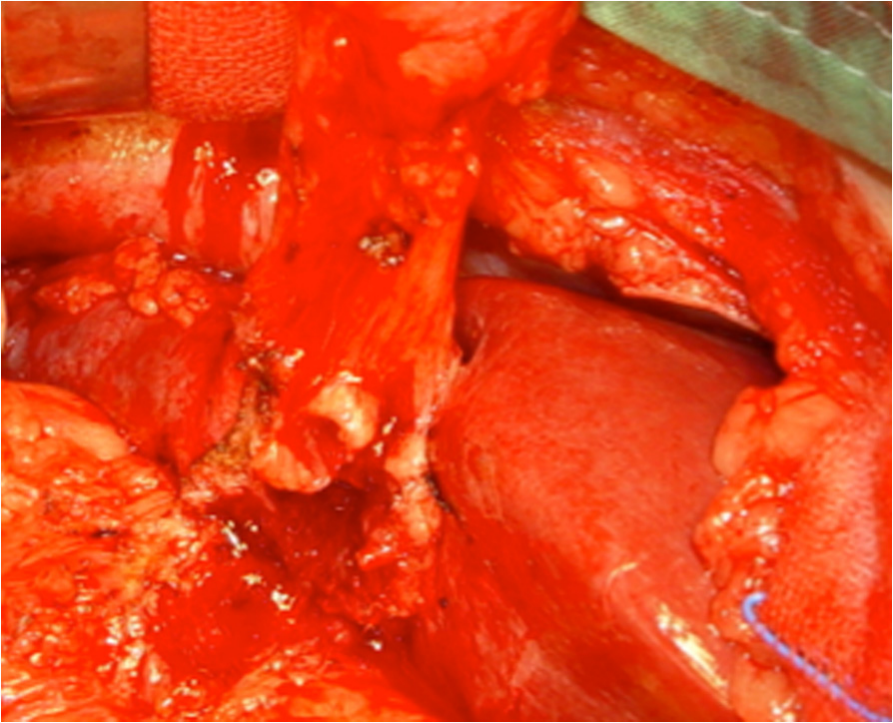
**The intra-operative finding before removing the gossypiboma.** Gossypiboma, about 2 cm in size, was noted between the supra-duodenal region and round ligament.

**Figure 5:**
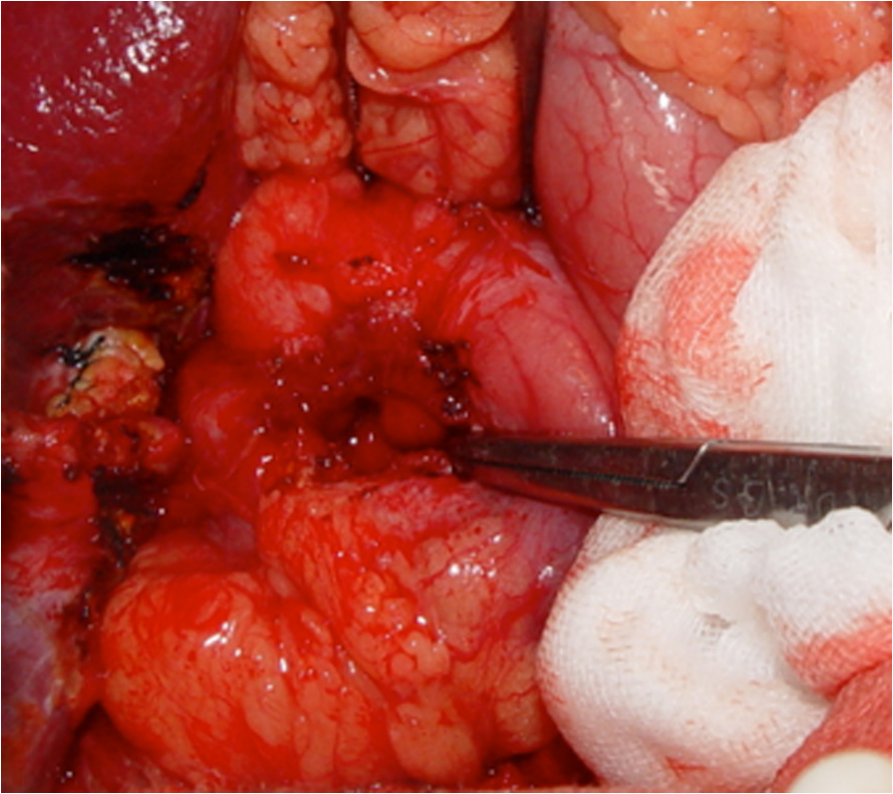
**The intra-operative finding after removing the gossypiboma.** A penetrating ulcer caused by the gossypiboma was noted over anterior wall of the duodenal bulb.

**Figure 6:**
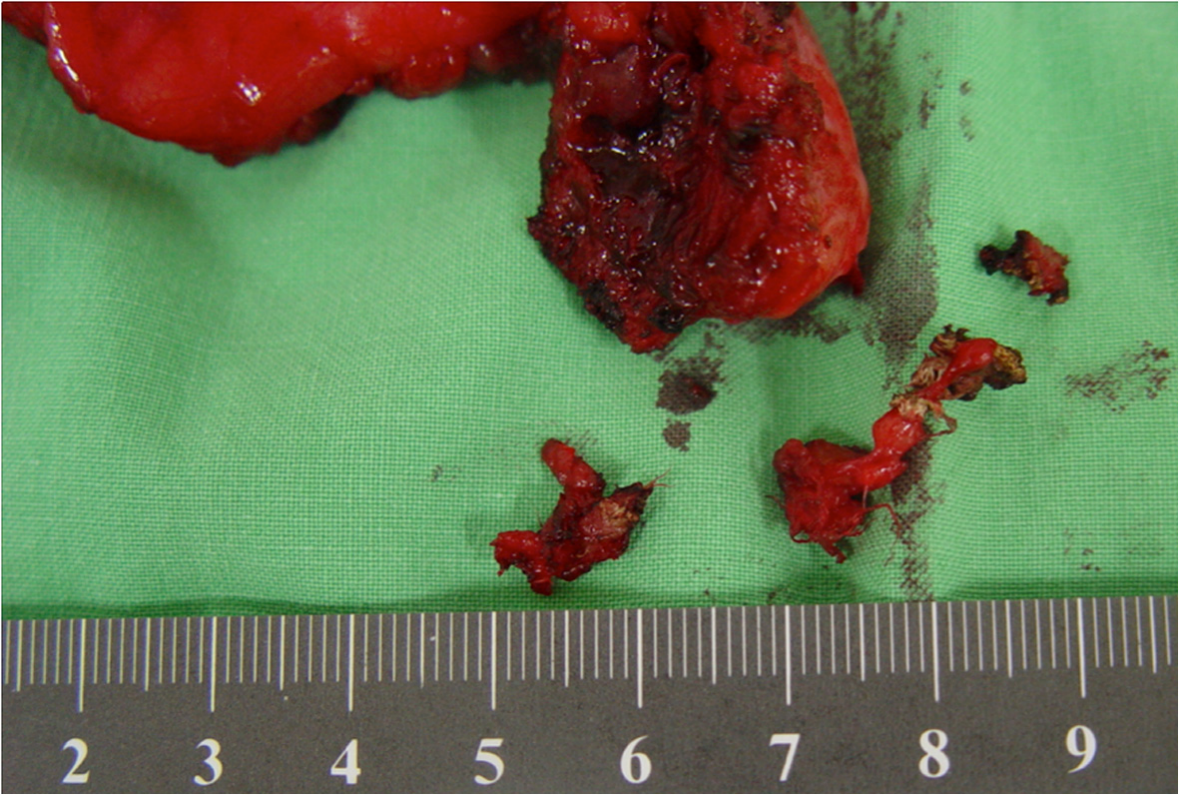
**The removed specimen.** The gauze was embedded in the granulation tissue surrounding the gossypiboma.

A 61-year-old Han Chinese man presented with intermittent epigastric pain for the last 10 months. The pain was mild and non-radiating, without specific relieving or aggravating factors. The patient had no history of nausea, vomiting, general weakness, poor appetite or body weight loss. He had undergone laparoscopic cholecystectomy conversion to open cholecystectomy for acute gangrenous cholecystitis 10 months ago at another hospital. Gauze retention in the peritoneal cavity with migration into the duodenum was noted after upper gastrointestinal (UGI) endoscopy (Figure 1). An abdominal X-ray examination showed the retained material was a surgical sponge (Figure 2). Abdominal computed tomography (CT) scan showed transmural migration of the gauze into the duodenum (Figure 3). Endoscopic intervention failed to remove the entire gauze, and intractable duodenal ulcer caused by the gauze persisted. Surgical intervention was then performed. During the operation, a gossypiboma, about 2 cm in size, was noted between the supra-duodenal region and round ligament (Figure 4), with penetration into the anterior wall of the duodenal bulb, resulting in a penetrated duodenal ulcer about 1.5 cm in diameter (Figure 5). The gauze was embedded in the granulation tissue surrounding the gossypiboma (Figure 6). The gauze was removed successfully, and the penetrated ulcer was repaired with duodenorrhaphy. The postoperative period was uneventful.

### Literature search

We searched the PubMed (2000–2013) database for case reports about transmural migration of gossypiboma into the duodenum. The abstracts of all articles published in Dutch, English, French, German, and Spanish were screened. The full texts of articles published in other languages but with an abstract in English were analyzed. Articles were selected for review if they included the following patient data: age, sex, initial surgery, interval, clinical presentation, diagnostic methods, location, and surgical procedures.

## Results

Our PubMed search yielded seven reports of transmural migration of retained surgical sponge into the duodenum. Relevant data are shown in Table 1. Three patients were male and four patients were female, with ages ranging from 26 to 62 years. The time from the causative operation to presentation with a retained surgical sponge ranged from 2 months to 2 years. Initial surgical procedures included cholecystectomy in 4 patients [5 patients, if the present case is included], laparotomy in 1, hemicolectomy in 1, and removal of hydatid cyst in 1 patient. Including our case, 3 cases were scheduled for open surgery. The other 4 patients were scheduled for endoscopy to remove the gossypiboma. One patient received endoscopy but the gauze could not be retrieved using biopsy forceps and a polypectomy snare. As there was no free perforation, conservative treatment was applied, and proton pump inhibitors and liquid diet were recommended. The patient had a stable clinical course and was endoscopically followed up at 5-day intervals.

**Table 1:** Transmural migration of gossypiboma into the duodenum: review of the selected literature (2000–2013)

**References**	**Initial surgery**	**Interval (months)**	**Clinical presentation**	**Diagnosis methods**	**Interventions (Surgical indication)**
Erdil et al. [2]	Cholecystectomy	12	GI bleeding	US,ERCP	Endoscopic extraction
Sinha et al. [4]	Laparotomy	12	Abdominal pain	US, CT, endoscopy	Endoscopic extraction
Alis et al. [5]	Hydatid cyst	2	Abdominal pain	Endoscopy, CT	Spontaneously expelled
Peyrin-Biroulet et al. [6]	Left, Hemicolectomy	6	Vomiting	Endoscopy, CT	Endoscopic extraction
Sarda et al.^7^	Cholecystectomy	2	Abdominal pain, vomiting	Endoscopy	Endoscopic extraction
Dux et al. [7]	Cholecystectomy	10	Abdominal pain, vomiting	CT	Surgical drainage (Persistent duodenal fistula)
Manikyam et al. [8]	Cholecystectomy	24	Abdominal pain, vomiting	US, Endoscopy	Right hemicolectomy, Duodenorraphy (Gastric outlet obstruction and duodeno-ileo-colic fistula)
Lv et al. (present study)	Cholecystectomy	10	Abdominal pain	Endoscopy CT	Endoscopic extraction, Duodenorrhaphy (Intractable duodenal ulcer)

## Discussion

The term “gossypiboma” denotes a cotton sponge that is retained inside a patient during surgery. The reported incidence of gossypiboma varies between 1/100 and 1/3000 for all surgical interventions and from 1/1000 to 1/1500 for intra-abdominal operations [9-18]. There are no national or local registers, and the reluctance of medical institutions to publish matters that may have medico-legal implications probably leads to underreporting of diagnosed cases. Furthermore, some patients remain asymptomatic and in such cases gossypibomas may never be found.

As a consequence of gossypiboma, two types of foreign body reactions can occur. The first type is an aseptic fibrous response to the foreign material that creates adhesions and encapsulation. The result is a foreign body granuloma which may take a silent clinical course which dose not produce any clinical symptoms. A gossypiboma may undergo calcification, disruption, partial absorption, and even diffusion. The second type of foreign body reaction is exudative in nature and produces an inflammatory reaction with abscess formation. The body attempts to extrude the foreign material, which may lead to post-surgical complications such as external fistula formation or erosion and perforation into adjacent viscera. This may then result in migration of the foreign body into the gut, intestinal obstruction, or extrusion of the sponge through the rectum. The exudative type of response often causes symptoms in the early postoperative period, but the extrusion process may take years and the clinical symptoms are unspecific [7-9,19-25]. Wattanasirichaigoon describes 4 stages in the process of migration: foreign body reaction, secondary infection, mass formation, and remodeling [25].

According to the literature, transmural migration of gossypiboma into the duodenum is rare. To date, only 7 cases have been published [2,4-8,26]. Other reported sites of migration include seven sponges into the jejunum, five into the stomach, five into the colon, one into the ileocolic region, one into the ileojejunal region, and one into both the jejunum and colon. In three patients, the surgical sponge passed spontaneously through the rectum. The small intestine is the most common part of the intestine into which migration takes place (Table 2). The most common initial surgery for removal of gossypiboma in the gastrointestinal system was cholecystectomy (15 cases), followed by caesarian section (9 cases), hysterectomy (7 cases), laparotomy (5 cases), appendectomy (3 cases), splenectomy (1 case), distal gastrectomy (1 case), hemicolectomy (1 case), cystectomy + myomectomy (1 case), hydatid cyst (1 case), nephrectomy (1 case) and anterior resection (1 case) (Table 3).

**Table 2:** Reported cases of transmural migration of gossypiboma into the gastrointestinal organs according to the impacted gastrointestinal organs: review of the selected literature (2000–2013)

**The impacted gastrointestinal organs**	**No. of patients**	**References**
Stomach	5	[9,19,27-29]
Duodenum	7	[2,4-8,26]
Small intestine	23	[7,9-14,19,20,27-45]
Colon	5	[15,46-49]
Small intestine and colon	3	[16,17,50]
Rectum	3	[1,18,48]

**Table 3:** Reported cases of transmural migration of gossypiboma into the gastrointestinal organs according to the initial procedures: review of the selected literature (2000–2013)

**Initial procedures**	**No. of patients**	**References**
Cholecystectomy	15	[2,7,8,12,14,19,27,29,30,36-39,43,47]
Caesarian section	9	[1,13,17,32-34,40,48,50]
Hysterectomy	7	[11,16,18,20,26,42,45]
Laparotomy	5	[4,15,20,44,49]
Appendectomy	3	[10,35,46]
Splenectomy	1	[12]
Distal Gastrectomy	1	[9]
Hemicolectomy	1	[6]
Cystectomy + Myomectomy	1	[41]
Hydatid cyst	1	[5]
Nephrectomy	1	[28]
Anterior resection	1	[28]

Many risk factors, such as duration and complexity of surgery, excessive blood loss in trauma patients, surgery under emergency conditions, unplanned procedural changes, a change in operating room teams during the course of the operation, and a failure to count surgical instruments and sponges, were identified. The three most important risk factors are emergency surgery, unplanned change in the operation, and body mass index [2,15,18,33].

Nonspecific clinical symptoms may preclude an accurate diagnosis. The clinical presentation of gossypiboma is variable. According to the literature, common symptoms and signs of transmural migration of gossypiboma into the duodenum may include abdominal pain, vomiting, and bleeding [7,9,30]. The most frequently reported symptom was abdominal pain. The main complications of abdominal gossypiboma were bowel or viscera perforation, obstruction, peritonitis, adhesion, abscess development, fistula formation, sepsis, and migration of the sponge into the lumens of the gastrointestinal tract [9,30].

The diagnosis of gossypiboma is difficult because the clinical symptoms are nonspecific and the imaging findings are often inconclusive. In imaging studies, they are mostly seen as radio-opaque material, yet radiolucent material like sponges can cause diagnostic problems. However, plain radiography, barium studies, endoscopy, ultrasonography (US), CT, and magnetic resonance imaging (MRI) are useful for diagnosis [17]. Plain radiographs may disclose the presence of gossypiboma if the surgical sponge is calcified or when a characteristic “whirl-like” pattern is evident. In the literatures, endoscopy played an important role in the diagnosis and treatment of intraluminal gossypiboma cases.

**Figure 7:**
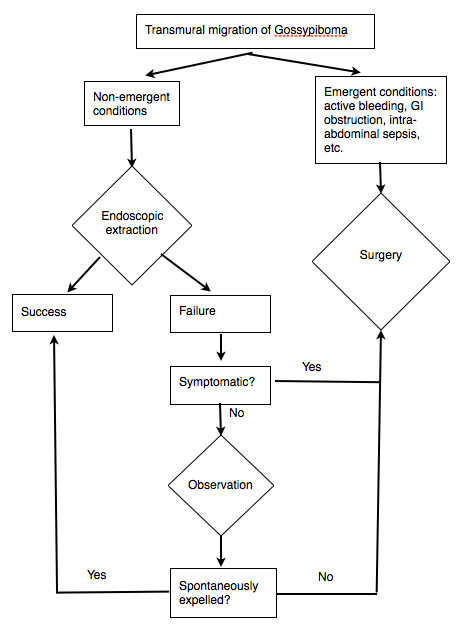
**Scheme of the therapeutic approach proposed for transmural migration of gossypiboma into gastrointestinal system.** For patients with gossypiboma transmurally migrated into gastrointestinal system, endoscopic removal could be attempted if there was no emergent conditions such as active GI bleeding, obstruction, or intra-abdominal sepsis. If the endoscopy fails to retrieve the gossypiboma and the patient is symptomatic, surgical intervention is indicated. For asymptomatic patients after unsuccessful endoscopic extraction, conservative treatment with close observation can be considered.

Gossypibomas should be removed as soon as diagnosed. Surgery is the preferred method of treatment for gossypiboma. Various techniques, including percutaneous techniques, such as laparoscopy and laparotomy, are used for the removal of gossypiboma, depending on the clinical presentation and medical equipment available [4,6,26,30,46]. In cases with migration of gossypiboma into the digestive tract, nonsurgical approaches such as endoscopic retrieval of foreign bodies have been reported. According to the literature, gastrostomy, segmental resection, and endoscopic extraction were used for removal of gossypibomas that migrated into the stomach, intestine and colon, and duodenum, respectively. For patients with gossypiboma transmurally migrated into duodenum, endoscopic removal could be attempted if there was no emergent conditions such as active GI bleeding, obstruction, or free perforation. Of the eight cases of gossypiboma migrated into duodenum, including our case, surgical intervention was necessary in three patients for persistent fistula or intractable ulcer. If the endoscopy fails to retrieve the gossypiboma and the patient is asymptomatic, conservative treatment with close observation can be considered. A therapeutic scheme is proposed for the treatment of gossypiboma transmurally migrated into the digestive tract (Figure 7).

## Conclusion

In conclusion, gossypiboma should be considered in the differential diagnosis of any postoperative patient who presents with pain, infection, or a palpable mass. Plain radiography, barium studies, endoscopy, ultrasonography, CT scan, and MRI are useful for diagnosis. Transmural migration of gossypiboma into the duodenum is a rare surgical complication. The treatment strategies include endoscopic removal and surgical intervention. Prompt surgical intervention should be considered for emergent conditions such as active bleeding, GI obstruction, or intra-abdominal sepsis. For non-emergent conditions, surgical intervention could be considered in intractable cases if endoscopic extraction failed.

## Consent

Written informed consent was obtained from the patient for publication of this Case report and any accompanying images. A copy of the written consent form is available for review by the Editor of this journal.

## Abbreviations

UGI: Upper Gastrointestinal; CT: Computed Tomography; US: Ultrasound; MRI: Magnetic resonance imaging; ERCP: Endoscopic retrograde cholangiopancreatography.

## Competing interests

The authors declare that they have no competing interests.

## Authors' contributions

YXL searched the literatures and drafted the manuscript. CCY conceived of the study, participated in its design and coordination, and final revision of the manuscript. CFT participated in the collection of the clinical data and design of the study. CCW participated in the design of the study and critical revision of the manuscript. All authors read and approved the final manuscript.

## Pre-publication history

The pre-publication history for this paper can be accessed here:

http://www.biomedcentral.com/1471-2482/14/36/prepub
